# Haloperidol for Pain Management: A Narrative Review

**DOI:** 10.3390/ph17081096

**Published:** 2024-08-21

**Authors:** Carlos J. Roldan, Jonathan W. Rowland, Alice L. Ye

**Affiliations:** 1 Department of Pain Medicine, The University of Texas MD Anderson Texas Center, Houston, TX 77030, USA; 2 McGovern Medical School, The University of Texas Health Science Center at Houston (UTHealth), Houston, TX 77030, USA; 3 Department of Emergency Medicine, The University of Texas MD Anderson Cancer Center, 1515 Holcombe Boulevard, Houston, TX 77030, USA

**Keywords:** haloperidol, pain, comprehensive, review, emergency, chronic

## Abstract

The use of haloperidol in pain management has been a topic of interest for several decades. Haloperidol is a widely used antipsychotic medication with unique pharmacologic properties that make it a potential candidate for pain management. However, the efficacy and safety of haloperidol for pain management remain controversial. This narrative review provides a summary of the current literature on the use of haloperidol for pain management, including its pharmacology, clinical effectiveness, adverse effects, and dosing regimens. We performed a comprehensive search of the literature for this review. The most robust clinical data from the past decade suggest that haloperidol has good efficacy in the treatment of pain related to gastroparesis and migraines and has shown promise for opioid use reduction in patients with chronic pain or receiving palliative care. The overall side effect profile is excellent, with zero reported events of QT-related cardiac arrest and minimal reports of sedation and transient extrapyramidal effects such as akathisia. Dosing regimens used were heterogeneous, with most ranging from 1 to 5 mg per dose via intravenous, intramuscular, or oral route. Studies with designs that isolated the effects of haloperidol from combinations of other drugs were extremely limited. Further high-quality prospective studies are needed to determine the ideal role of haloperidol in the routine clinical management of painful conditions.

## 1. Introduction

Simple but ingenious methods of animal pharmacology have exposed the unique analgesic properties of medications not considered to be analgesics. A sui generis example is haloperidol, a first-generation antipsychotic that exerts its main action by the nonselective blockade of dopamine D2 receptors in the brain [1]. Among other mechanisms, haloperidol has noradrenergic, cholinergic, and histaminergic blocking action (Table 1). The blocking of these receptors is potentially associated with various effects, including analgesia [2]. Haloperidol was synthesized in 1958 by Bert Hermans at the Janssen Laboratories, Beerse, Belgium, and was initially called R1625, then haloperidol [3]. The first medical publication on haloperidol described its neuroleptic properties that have kept haloperidol as one of the first-generation antipsychotics worldwide 40 years after its discovery [4]. US Food and Drug Administration-approved use of haloperidol includes for the treatment of schizophrenia, Tourette syndrome, and some behavioral disorders in children [5]. Off-label uses of haloperidol have been explored in various clinical settings for pain management.

Although haloperidol has been referenced as a potential analgesic for a variety of pain conditions, current data do not support its effectiveness as a first-line option, but rather as an essential adjuvant agent when other pain treatments fail or used as an adjunct to minimize opioid burden. A good but limited number of clinical trials present haloperidol as a first analgesic agent in acute gastroparesis and migraines. Thus, the aim of this review is to explore the use of haloperidol for pain management, including its mechanisms, pharmacology, clinical indications, adverse effects, and common doses.

## 2. Material and Methods

### Literature Search

A comprehensive literature search was constructed using the following terms: haloperidol/or *haloperidol/pain, (haloperidol or Haldol), (pain* or neuropathy or emergency). PubMed, Medline (Ovid), Embase (Ovid), Scopus, and Google Scholar were queried from 2013 through November 2023, using controlled vocabulary terms for “haloperidol” and “pain”.

**Figure 1:**
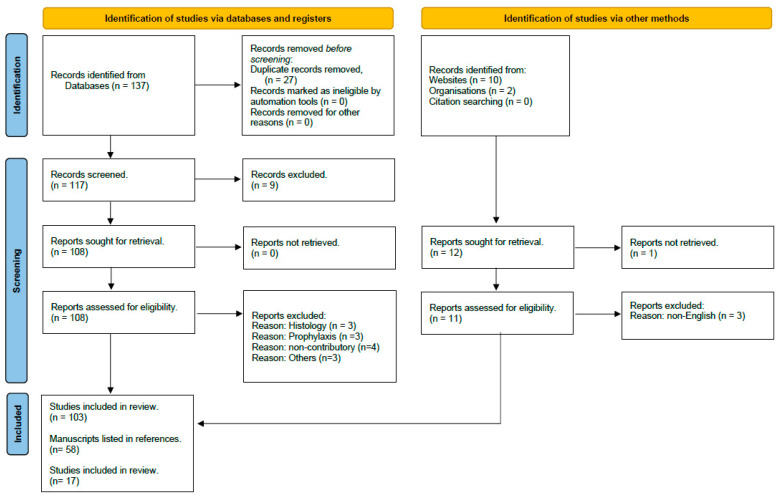
PRISMA 2020 flow diagram.

The search yielded 137 articles including animal studies; in the appraisal approach adopted, papers that were not available on the databases searched were not included. The results were limited to new (within the past 10 years) and highly cited references published in English; conference abstracts were eliminated, while bibliographies of articles discovered for additional relevant literature works were also examined. We included a few other relevant articles cited in the selected manuscripts. After de-duplication, 103 unique records were identified and included (Figure 1). Each title was reviewed, with its abstract, if available, to ascertain its relevance. Data were extracted from each study report to allow for a comparison of interventions and to assess the quality of study designs. The characteristics chosen for comparison included the type of study, size, methodology, outcome measures, results, and conclusions. The highest-quality rating scale was double-blinding as the central methodology; while not all trials could be blinded, randomization in non-blinded studies was overall higher valued than observational studies or expert opinions.

The aims, data search, and synthesis, interpretation, and recommendations were the result of unanimously approved verbal and written contributions from the convening of the authors.

## 3. Discussion

### 3.1. Pharmacokinetics and Pharmacodynamics

Haloperidol is a well-absorbed medication if provided via the enteral route, but its bioavailability decreases to 40–75% after first-pass hepatic metabolism. Serum concentrations of haloperidol peak at 0.5–4 h after absorption from the gastrointestinal tract. Its estimated volume of distribution ranges from 11 to 25 L/kg, which reflects a high degree of lipophilicity and suggests extensive, steady-state extravascular localization [15]. Haloperidol is predominantly (90–94%) bound to plasma proteins when circulating in the blood. Renal excretion of haloperidol is minimal to negligible; clearance occurs almost entirely by hepatic metabolism [16]. To date, only one active metabolite of haloperidol has been identified, reduced haloperidol, which is produced by the hydroxylation of haloperidol and retains only about 10% of the pharmacologic activity of the parent drug. In patients with normal liver function, the elimination half-life of parental haloperidol is 13–35 h [17].

Three enzymes are involved in the biotransformation of haloperidol: cytochrome P450 (CYP), carbonyl reductase, and uridine diphosphoglucose glucuronosyl transferase (UDGT). The initial process includes glucuronidation conducted by UDGT, followed by CYP-mediated oxidation. In vitro studies show that CYP3A4 is the main isoform responsible for its metabolism in humans [18]. The catalytic activity of CYP-mediated reactions has a wide range; although this catalytic activity does not affect the glucuronidation and carbonyl reduction pathways, and the clinical implications are unclear. However, pharmacologic studies have shown that interactions of haloperidol with most drugs lead to only minor changes in plasma concentrations of haloperidol, suggesting that these interactions have little clinical significance [19].

Studies analyzing drug interactions have suggested that because CYP3A4 is involved in the biotransformation of haloperidol, pharmacokinetic interactions might occur when various drugs are given concomitantly. For instance, medications such as carbamazepine, phenytoin, phenobarbital, venlafaxine, buspirone, alprazolam, rifampicin, St John’s Wort, and carteolol are CYP450 inducers, resulting in the increased metabolism of haloperidol and subsequently reducing its therapeutic concentration [20]. Conversely, co-administration of enzyme CYP450 inhibitors such as alprazolam, ketoconazole, itraconazole, ritonavir, nefazodone, chlorpromazine, promethazine, paroxetine, quinidine, sertraline, venlafaxine, fluvoxamine, fluoxetine, and ritonavir may increase the serum concentration of haloperidol, requiring special monitoring or dose reduction [21].

### 3.2. Analgesic Mechanisms

The analgesic properties of haloperidol have been studied using various approaches and animal models. Thus, various mechanisms of antinociception have been suggested, as summarized in Table 1.

The dopaminergic system and its underlying mechanisms in pain remain unclear. In an animal migraine study, apomorphine-mediated dopaminergic activation exacerbated nitroglycerin-stimulated nociceptive reactions, CGRP release, and mast cell degranulation. Haloperidol, a D2 receptor antagonist, was effective in reducing those migraine-related parameters and migraine-like conditions [6]. Likewise, by blocking the dopaminergic system, haloperidol changed the habituation to absolute pain over time despite a linear increase in intensity [7]. In a placebo analgesia animal model aiming to measure the response to high-level pain, cue preference was mediated by reward learning via blockade of the dopamine system with haloperidol [8].

The sigma-1 receptor is a unique ligand-operated chaperone present in crucial areas for pain control in both the peripheral and central nervous systems [9]. Haloperidol antagonizes the binding of the sigma-1 receptor to the NR1 subunits of NMDA receptors activated by a ligand, potentiating opioid-induced antinociception. This opened the possibility of using haloperidol, a sigma-1 receptor antagonist, in clinical practice as an opioid adjuvant [22]. A similar effect was obtained in an experimental model of neuropathic pain, which corroborated the antagonistic action of the sigma-1 receptor, using gabapentin as a positive control [10].

N-methyl-d-aspartate (NDMA) channels are implicated in the induction and maintenance of peripheral and central sensitization during nociceptive states. Thus, selective NMDA channel antagonism, which has been reproduced with haloperidol, has been associated with analgesia in a free-moving state without loss of the righting reflex [11].

Mu receptor synergism has been proposed as the reason why haloperidol enhanced morphine analgesia when the two drugs were co-administered [12]. Likewise, haloperidol enhanced the antinociceptive effect of morphine because haloperidol was able to disrupt or delay morphine tolerance in neuropathic pain [14].

The zona incerta, a subthalamic nucleus, is connected to several structures involved in both antinociception and nociception. Glutamate-induced stimulation of the zona incerta in rats treated with haloperidol was shown to activate a pain-inhibitory mechanism that descends to the spinal cord via the dorsolateral funiculus [14].

Other authors have described the effects of haloperidol on additional nociceptive pathways, including 5-HT2 serotonin, alpha adrenergic, H1 histamine, and muscarinic receptors in the brain [23]. Similarly, because haloperidol has structural similarities to meperidine, loperamide, and diphenoxylate, some have proposed a peripherally limited mu-opioid agonist effect [3,24].

## 4. Review of Clinical Studies

### 4.1. Haloperidol for Acute Emergency Care Pain Conditions

We found 11 studies that assessed haloperidol as a single agent or combined with other agents for acute pain in the emergency care setting (Table 2). Nine of these studies compared haloperidol with another pharmaceutical agent or placebo. Only three trials were placebo-controlled [25,26,27]. Six studies involved specific pain conditions (gastroparesis, renal colic, migraine, and moderate to severe burns), and the other five studies involved more heterogenous conditions (acute pain, abdominal pain, and headache).

Two studies examined haloperidol for the treatment of acute gastroparesis. Ramirez et al. retrospectively examined patients with self-matched emergency department (ED) encounters. They reported patients receiving 5 mg intramuscular haloperidol, observing a small decrease in total opioids used (equivalent to about 40 mg tramadol or 2.5 mg oxycodone) and a modest decrease in hospital admissions (−17%), compared with no haloperidol [28]. Roldan et al. performed a placebo-controlled double-blinded randomized trial and found a substantial reduction in reported pain (−4.0-point difference compared with the placebo using a pain visual analog scale ranging from 0 to 10) and nearly half as many hospital admissions in patients who received 5 mg intravenous haloperidol compared with the placebo (−46%) [25]. This difference in pain scores is greater than accepted clinically significant differences reported for musculoskeletal pain (≥2-point reduction on a pain scale ranging from 0 to 10) and acute pain in ED settings (≥3-point reduction on a pain scale ranging from 0 to 10) [42,43]. Mechanistically, the pain relief seen with haloperidol could be mediated through gastrointestinal D2 receptor blockade, thus preventing acetylcholine release and the downstream slowing of stomach motility [44].

Masoumi et al. examined whether 5 mg haloperidol as an adjunct to morphine was beneficial for acute renal colic [29]. They found no difference in pain scores or in the incidence of nausea and vomiting; however, another study by Kazemi et al. examining postoperative pain control in opioid users noted that a high dose of 20 mg haloperidol combined with morphine was more effective for short-term pain relief than morphine alone [29,37]. Perhaps a higher dosage of haloperidol is needed for pain relief in acute pain that is primarily due to a prostaglandin-driven inflammatory response. However, a dose of 5 mg or greater of haloperidol can risk torsades de pointes and potential cardiac arrest [42,45,46]. Clinically, this risk likely outweighs any benefit of high-dose haloperidol as an acute inflammatory pain adjunct.

Two studies looked at nonspecific acute abdominal pain, including undiagnosed irritable bowel syndrome, celiac disease, gynecologic causes, or even malignancies [30,31,47]. Heard et al. performed a retrospective cohort study on a large sample across 18 sites and found that patients who received any haloperidol for pain had a 1.4 relative risk increase for receiving intravenous opioids, also interpreted as a 13% risk difference based on available tabular data [30]. It is unclear if the patients who were not exposed to haloperidol received other non-opioid pain medications. Additionally, the dosages of haloperidol used in exposed encounters were not reported. Knudsen-Lachendro et al. retrospectively examined patients with self-matched ED encounters, comparing haloperidol with opioids for the treatment of nonspecific abdominal pain [31]. They reported that, overall, encounters with haloperidol resulted in a mild decrease in total opioid use, a mild decrease in rescue anti-emetic use (−14%), and a moderate decrease in rescue analgesia use (−24%); however, these results were confounded by analysis showing that there was 25% more ketorolac use in the haloperidol use encounters than in the opioid use encounters and that 32% of events categorized as haloperidol use encounters were in patients who also received opioids. The data analysis approach may have benefited from a post hoc stratified or multivariate analysis to control for confounders such as opioid and ketorolac use [48], given that heterogeneous causes of pain with different pain pathways were clustered under a nonspecific clinical syndrome.

Two randomized trials looked at haloperidol with another agent for any acute pain, including trauma-related pain [32,33]. Moradi et al. studied, in participants without substance use disorders, the combination of 2.5 mg haloperidol and weight-dosed ketamine compared with weight-dosed fentanyl for pain [32]. Afzalimoghaddam et al. studied, in opium users with acute pain, the combination of 2.5 mg haloperidol with 2.5 mg midazolam and weight-dosed morphine compared with weight-dosed morphine alone [33]. Both studies showed a greater decrease in pain scores in the intervention arm over the comparison arm in the short term (30 min to 1 h). Unfortunately, it was difficult to interpret how much pain relief was due to haloperidol alone, given the multiple agents used in both studies. It has previously been shown that intranasal ketamine alone is noninferior to intranasal fentanyl, so the Moradi et al. trial design may have benefited from an additional arm of ketamine alone to assess whether haloperidol plus ketamine added any additional benefit [49]. It remains unclear whether midazolam more than haloperidol may have been primarily responsible for the outcomes observed.

Three randomized trials assessed acute migraines, with one of the three trials including non-migraine headaches as well [26,27,34]. Gaffigan et al. compared diphenhydramine and haloperidol with diphenhydramine and metoclopramide, finding no difference in pain scores at 80 min but a decreased rate of rescue analgesic use in the haloperidol arm (−21% difference) [34]. It is unclear why rescue analgesic use decreased more in the haloperidol arm, but pain scores were similar. Both arms reached maximum pain relief at less than 1 h after treatment. What may have helped to clarify this could have been tracking whether the participants in the haloperidol arm had a quicker onset of pain relief than the metoclopramide arm and thus less need overall for rescue analgesics. Kazemi et al. also noted that the analgesic effects of haloperidol are likely short-lived [34].

Honkaniemi et al. and McCoy et al. both conducted randomized placebo-controlled trials using intravenous haloperidol [26,27]. Honkaniemi et al. assessed 5 mg haloperidol in a migraine-only sample; McCoy et al. assessed 2.5 mg haloperidol in all headaches, including migraines. Both studies showed significant decreases in pain scores compared with the placebo, and the higher dose of haloperidol appeared more effective. Like gastroparesis pathogenesis, D2 receptor activation has also been implicated in migraine development, and central D2 receptor blockade by haloperidol likely contributes to the pain relief seen in these trials [49]. However, none of the three trials used the primary endpoints recommended by the International Headache Society: pain freedom and absence of most bothersome symptoms at 2 h [50].

The last study of haloperidol for acute pain was by Ali et al., who conducted a single-arm prospective trial of oral weight-dosed haloperidol (e.g., about 4.5 mg to 13.5 mg for a 91 kg adult) with 100–200 mg carbamazepine and with 300–400 mg intravenous tramadol as a 12 h infusion for burn patients [35]. Although pain and pain-related behaviors decreased across the 7-day trial, it is unknown how much of this benefit was attributable to haloperidol given the two other pain-modulatory agents. Of concern, too, is the combination of high-dose tramadol with carbamazepine, a contraindicated combination, because carbamazepine can reduce tramadol efficacy and increase the risk of serotonin syndrome [51]. Pain from severe burns is challenging to treat and often requires a multimodal pharmaceutical approach involving opioids. To assess whether haloperidol plays an important adjunctive role, future trials should consider comparative trial designs, making sure to capture differences in rescue analgesic use [52].

### 4.2. Haloperidol for Acute Postoperative Pain Conditions

Four studies examined haloperidol for postoperative pain and nausea control (Table 2). Postoperative nausea is common after surgery, with increased risk if patients are given postoperative opioids [53]. Several guideline-based preventative anti-emetic agents exist, including low-dose (0.5–2 mg) haloperidol. However, haloperidol is not listed as a recommended agent for postoperative pain control [54]. The four trials reviewed haloperidol with and/or against combinations of dexamethasone, morphine, ondansetron, and diazepam.

Judkins and Harmer in 1982 assessed 5–10 mg intravenous haloperidol with premedication diazepam compared with the placebo with premedication diazepam for postoperative pain and nausea in patients undergoing elective major abdominal surgery [39]. They found no difference in pain scores at 24 h, a mild increase in morphine milligram equivalents in patients who received haloperidol, and a decrease in nausea scores for haloperidol compared with the placebo when combined with diazepam. The proper study conclusion should be that haloperidol can be a useful adjunctive with diazepam for nausea, but not pain, by 24 h.

Benevides et al. in 2013 conducted a comparative trial of three arms assessing 2 mg intravenous haloperidol combined with dexamethasone and ondansetron in patients undergoing laparoscopic gastrectomy. This combination was mildly to modestly more effective for pain and mildly more effective for nausea than ondansetron alone at 2 h after surgery [38].

Two years later, Kazemi et al. published the results of a placebo-controlled trial examining 20 mg intravenous haloperidol with morphine compared with morphine alone in opium users undergoing orthopedic surgery. In that study, haloperidol as an adjunct to morphine resulted in a moderately decreased pain score difference by 30 min but showed similar pain scores to those of morphine alone by 2 h [37]. This trial design is more aligned with showing haloperidol as a useful adjunctive pain agent.

Heriwardito et al., in 2022, assessed 1 mg intravenous haloperidol compared with 5 mg intravenous dexamethasone in patients undergoing elective laparoscopic surgery [36]. Although ideally both interventions should be given at the same surgical timepoint, guideline-based recommendations note that haloperidol can be given both at induction and before the end of surgery with equivalent results [53]. The results of this trial showed that haloperidol led to a decrease in pain and reported incidence of nausea compared with dexamethasone at both 6–12 and 12–24 h. The study team attributed this dramatic difference to the shorter duration of action of dexamethasone (12 h) compared with haloperidol (24 h).

Across the mentioned studies, there is a general trend that haloperidol may have some benefit against postoperative pain and nausea; however, the patient populations and interventions studied varied widely, making it difficult to provide a summary statement about haloperidol.

### 4.3. Haloperidol for Chronic Pain Conditions

We reviewed two studies that assessed daily haloperidol for chronic pain (Table 2). A 1979 study by Raft et al. was a prospective single-arm trial in a very small sample assessing haloperidol with multimodal pain management techniques for chronic facial pain [41]. They identified patients who were previously resistant to relaxation techniques. All 16 patients reported pain improvement, although only 12 successfully completed the relaxation protocol. It is unclear how they translated the Tourniquet pain assessment test results to pain improvement.

Salpeter et al. in 2015 retrospectively assessed very low-dose daily haloperidol with methadone for patients receiving inpatient palliative care [40]. They compared pain scores and morphine-equivalent daily dose outcomes between one arm that received haloperidol for breakthrough pain and another arm that received short-acting opiates for breakthrough pain. The study team found that by 2 weeks, those who received haloperidol for breakthrough pain reported modestly lower pain scores and fewer incidents of severe pain than those in the opiate arm. The morphine-equivalent daily dose significantly dropped in both arms, with a greater decrease in the haloperidol arm than in the opiate arm. This was the only study of the 17 identified that also examined haloperidol for pain in cancer patients. Pain scores decreased significantly over the intervention period for both cancer and noncancer participants, but cancer participants had mildly increased final peak pain scores across both arms when compared with noncancer participants. Additionally, while noncancer participants’ median pain scores were 0 by week 2, cancer participants continued to have a median pain score of 2.3.

More promising is the evidence for very low-dose haloperidol as an adjunct to methadone for lowering opioid burden and improving pain control in palliative care.

### 4.4. Incidence of Adverse Effects with Haloperidol

Many adverse effects are associated with haloperidol use because of its nonselective properties. The most common effects are extrapyramidal events such as akathisia (sensation of restlessness) [5]. Rarely, in patients with risk factors for prolonged QT intervals, haloperidol can induce torsades de points and, even more rarely, cause sudden cardiac arrest [55,56]. A review of past case reports noted that QT prolongation can occur at doses ranging from 2 mg to 1540 mg, and torsades de pointes can occur at doses ranging from 5 mg to 645 mg [55]. In many patients, polypharmacy involving other agents with a similar effect on the QT segment can mistakenly attribute these events to haloperidol alone. An early systematic review noted that extrapyramidal effects from haloperidol may be dose-dependent, but a recent update to that systematic review noted no increased risk of such a side effect.

In our review, 13 of the 17 studies reported on adverse events associated with haloperidol use. Six of these studies found no side effects, with haloperidol doses ranging from 1 mg to 20 mg [25,28,31,33,36,37]. Three studies included cardiac monitoring for the duration of the intervention and found no significant changes in QT lengths in haloperidol dosing of 2.5 mg to 5 mg [25,27,34]. Of the seven studies that noted adverse events (Table 3), the most consistent findings were extrapyramidal-related effects such as reported restlessness, motor agitation, and stiffness/shaking; less often seen was sedation. There were no reports of acute dystonic episodes. Raft et al. [41] reported the longest duration of haloperidol use, up to 6 weeks, and noted no extrapyramidal effects; however, a more recent case report described the development of tardive dyskinesia following 3 mg of daily haloperidol for 7 months [57].

All three migraine trials had follow-up data [26,27,34]. Honkaniemi et al. noted that seven patients in the haloperidol arm had recurrence of migraines and that seven patients reported that they would not like to be treated with haloperidol in the future [26]. It is difficult to interpret these findings because no comparative placebo data were given. Gaffigan et al. conducted a follow-up between 48 h and 2 weeks, with a 67% response rate, showing that 12% fewer participants in the haloperidol arm had migraine recurrence (48% compared with 60%) and 0% returned to the ED due to migraine attack [34]. Patients in both arms were highly satisfied with treatment (91% in the haloperidol arm and 89% in the placebo arm). However, haloperidol participants reported 33% more restlessness, 12% more sleepiness, and 7% more agitation. McCoy et al. [27] had a 95% response rate for 24 h follow-up, reporting that 18% fewer participants in the haloperidol arm had headache recurrence (33% compared with 51%), 10% fewer participants returned to the ED for additional care (7.2% compared with 17.5%), and 41% more participants requested haloperidol for future care compared with the placebo (76% compared with 35%). In sum, most participants appeared satisfied with its use for migraines and headaches.

### 4.5. Future Directions

The search for the ideal analgesic is an elusive task. Therefore, there is a great need to identify more treatment modalities for pain of any nature. Ideally, the treatment should be noninvasive, safe, efficient, and cost-effective, while providing sustained analgesia. Thus, atypical analgesics are tried, yielding mixed results. Haloperidol’s analgesic properties have been explored in different clinical scenarios, at times with unexpected high efficiency and safety.

Perhaps good patient selection and pathology are key for good outcomes.

Although some published studies have reported good results, ideally, multicenter, randomized, placebo-controlled studies can address any introduced bias. Furthermore, this can validate haloperidol’s safety and efficiency, particularly when used in combination with other agents.

### 4.6. Limitations

Enhanced reporting systems that streamline data collection from diverse sources, including healthcare providers, patients, and registries, were not part of this review to help obtain a better understanding of the role of haloperidol in pain management. Furthermore, inadequacies in data reported in retrospective and observational studies such as patient compliance and the introduction of co-intervention bias could not be addressed owing to their retrospective nature. Some randomized controlled studies with small sample sizes might have introduced type I error which may be unmasked in larger studies. Finally, while conventional analgesics were used in comparison groups, it was difficult to attribute the reduction in symptom intensity to haloperidol alone.

## 5. Conclusions

Haloperidol has recently gained more attention for its potential analgesic effects. There is a growing understanding of its analgesic mechanisms, which appear to be wide-ranging, including dopamine receptor blockade, NMDA pathway modulation, possible mu receptor synergism, and more. A limited number of clinical trials have shown that haloperidol, either alone or in combination with another analgesic, may be beneficial for a variety of pain conditions, with favorable research supporting its use in acute gastroparesis and migraines. Most trials have involved low to very low doses of haloperidol, without findings of QT prolongation but with notable rates of restlessness and sedation after treatment. Given its risk profile and the existence of more targeted analgesic treatments for most conditions, low-dose haloperidol is unlikely to be a first-line pain treatment but may be an essential secondary agent when other pain treatments have been unsuccessful or employed as an adjunct to minimize opioid burden.

## Figures and Tables

**Table 1:** Proposed analgesic mechanisms for haloperidol, based on animal studies.

Study	Mechanism Proposed	Mechanism Detail	Model of Research
Baranoglu et al., 2023 [6]	Antagonism of D2 receptor	Blockade of CGRP release and mast cell degranulation	Migraine
Bauch et al., 2017 [7]	Antagonism of D2 receptor	Facilitation of habituation to the absolute pain intensity	Brain models
Lee et al., 2015 [8]	Antagonism of D2 receptor	Haloperidol blocked the place preference effect	Conditioned place preference and hot plate test
Deciga-Campos et al., 2021 [9]	Blockade of sigma-1 receptors	Anti-allodynic and anti-hyperalgesic activity; efficacy like gabapentin; potency two times higher	In vitro binding assay
Espinosa-Juarez et al., 2017 [10]	Blockade of sigma-1 receptors	Antinociceptive effect at the spinal level	Chronic constriction injury
Kikuchi et al., 2015 [11]	Blockade of NMDA channels	Could induce pain suppression without anesthesia	Loss of the righting reflex
Leppert et al., 2014 [12]	Synergism of mu receptors	Enhanced morphine analgesia	Tail-flick test
Mena-Valdes 2021 [13]	Synergism of mu receptors	Antagonized morphine tolerance in neuropathic pain	Chronic constriction injury
Petronilho et al., 2012 [14]	Stimulation of the zona incerta	Activation of a pain-inhibitory mechanism to the spinal cord	Tail-flick test and a rat model of incision

**Table 2:** Studies of haloperidol for various pain conditions (17 studies total).

Study	Indication	Design	Study Size (Female/Total %)	Excluded Populations ^a^	Intervention Arm	Comparison Arm	Key Results, Intervention vs. Comparison	Other Results, Intervention vs. Comparison	Adverse Events Reported
Acute emergency care (11 studies)									
Roldan et al., 2017 [25]	Acute gastroparesis	Randomized controlled trial	33 (73%)	Prolonged QT intervals	IV 5 mg haloperidol	IV placebo	−4.0 pain VAS (0–10) mean diff. at 1 h (*p* = NR)	−46% hospital admission diff. (*p* = 0.009); −1.6 nausea VAS (0–5) mean diff. at 1 h (*p* = NR)	I = 0/37 C = 0/37
Honkaniemi et al., 2006 [26]	Acute migraine	Randomized controlled trial	40 (unknown)	Prolonged QT intervals; psychiatric conditions	IV 5 mg haloperidol in normal saline	IV normal saline alone	−4.0 pain VAS (0–10) mean diff. at 1 to 3 h (*p* < 0.0001)	65% more patients reported “marked relief” (*p* < 0.0001)	I = 16/20 C = 1/20
McCoy et al., 2020 [27]	Acute headache or migraine	Randomized controlled trial	118 (73%)	Prolonged QT intervals	IV 2.5 mg haloperidol in normal saline	IV 5 normal saline alone	−2.9 pain VAS (0–10) mean diff. at 1 h (*p* unclear)	−47% less rescue analgesic needed (*p* = NR); +42% more reported ≥50% pain relief at 1 h (*p* = NR)	I = 14/58 C = 5/60
Ramirez et al., 2017 [28]	Acute gastroparesis	Retrospective comparative study	52 (62%)	--	IM 5 mg haloperidol	Self-matched to an ED visit without haloperidol	−4.0 MME median diff. between encounters (*p* < 0.009)	−17% hospital admission diff. (*p* < 0.02); no difference in additional antiemetics given (*p* = NR)	I = 0/52 C = 0/52
Masoumi et al., 2019 [29]	Acute renal colic	Randomized comparative trial	140 (29%)	Substance use disorders	IV 5 mg haloperidol in normal saline + 5 mg morphine	IV normal saline + 5 mg morphine	No difference in pain VAS (0–10) at 1 h (*p* = 0.38)	No difference in incidence of nausea and vomiting (*p* = 0.40, *p* = 0.61)	I = 73/140 C = 72/140
Heard et al., 2020 [30]	Nonspecific acute abdominal pain	Retrospective cohort study	11,688 (67%)	--	haloperidol used during ED encounter (dose NR)	No haloperidol used during ED encounter	RR 1.4 (95% CI 1.2–1.6) increase in IV opioid use	--	NA
Knudsen-Lachendro et al., 2021 [31]	Nonspecific acute abdominal pain	Retrospective comparative study	107 (70%)	Chronic haloperidol use	IM or IV 2–5 mg haloperidol (median 5 mg)	Self-matched to an ED visit with opioids	−5.7 MME median diff. (*p* < 0.001), confounded by +25% more ketorolac use in intervention arm	−24% less rescue analgesic needed (*p* < 0.001); −14% less rescue anti-emetic needed (*p* = 0.05)	I = 0/107 C = 1/107
Moradi et al., 2022 [32]	Acute pain (47% trauma)	Randomized comparative trial	200 (31%)	Psychiatric conditions; chronic pain; substance use disorder	IV 2.5 mg haloperidol + 0.3 mg/kg ketamine	IV 1 µg/kg fentanyl	−2.0 pain NRS (0–10) mean diff. at 30 min (*p* = NR); +56% more reported painless by 10 min (*p* < 0.001)	No difference in Richmond Agitation-Sedation Scale (mean for both was 0, *p* = NR)	I = 9/200 C = 2/200
Afzalimoghaddam et al., 2016 [33]	Acute pain in opium users (% trauma unknown)	Randomized comparative trial	87 (22%)	Prolonged QT intervals	IV 50 µg/kg morphine + IM 2.5 mg midazolam + 2.5 mg haloperidol in 5 mL distilled water	IV 50 µg/kg morphine + IM 5 mL distilled water	−1.2 pain NRS (0–10) mean diff. at 1 h (*p* = 0.001); −0.4 pain NRS mean diff. at 6 h (*p* = 0.05)	−12 additional MME given (*p* = 0.02)	I = 0/87 C = 0/87
Gaffigan et al., 2015 [34]	Acute migraine	Randomized comparative trial	64 (81%)	Prolonged QT intervals; heart disease; other neurologic conditions	IV 25 mg diphenhydramine + 5 mg haloperidol	IV 25 mg diphenhydramine + 10 mg metoclopramide	No difference in pain VAS (0–100) mean diff. at 80 min (*p* > 0.05)	No difference in nausea, restlessness, and sedation VAS (0–100) scores (*p* > 0.05); −21% less rescue analgesic needed (*p* < 0.02)	I = 17/64 C = 14/64
Ali et al., 2018 [35]	Acute burn 15–40% body surface area	Prospective single-arm trial	30 (unknown)	Hypertension, renal or hepatic impairment	PO 0.05–0.15 mg/kg haloperidol daily + PO 100–200 mg carbamazepine twice/day + IV 300–400 mg tramadol 12-h infusion	--	−6.7-point mean decrease from day 1 to 7 on a Wong-Baker Faces Pain Rating Scale (0–10 range) (*p* < 0.001)	−6.7-point mean decrease from day 1 to 7 on study team’s self-developed pain behavior scale (0–20) (*p* = NR)	I = 0/30 C = 0/30
Acute postoperative pain (4 studies)									
Heriwardito et al., 2022 [36]	Elective laparoscopic surgery	Randomized comparative trial	80 (65%)	Psychiatric and neurologic conditions	IV 1 mg haloperidol 1 h before end of surgery	IV 5 mg dexamethasone after induction	−1.5 pain VAS (0–10) mean diff. at 6–12 and 12–24 h (*p* < 0.001 both)	−15% less nausea at 6–12 h; −48% less nausea at 12–24 h (*p* < 0.02 both); no difference in vomiting	I = 0/80 C = 0/80
Kazemi et al., 2015 [37]	Elective orthopedic surgery in opium users	Randomized controlled trial	101 (0%)	Psychiatric conditions	IV 0.1 mg/kg morphine + 20 mg haloperidol + normal saline	IV 0.1 mg/kg morphine + normal saline	−2.7 pain Likert scale (0–4) ^b^ mean diff. at 30 min; −0.1 mean diff. at 2 h (*p* = NR)	−8.0 MME mean diff. in extra analgesic use (*p* < 0.001)	I = 0/101 C = 0/101
Benevides et al., 2013 [38]	Elective laparoscopic sleeve gastrectomy	Randomized comparative trial	90 (68%)	Psychiatric conditions; prior opioid use	IV 2 mg haloperidol + 8 mg dexamethasone + 8 mg ondansetron	IV 8 mg dexamethasone + IV 8 mg ondansetron ^c^	−1.5 pain NRS (0–10) mean diff. at 2 h for ondansetron only (*p* = 0.05); −1.9 pain NRS mean diff. for ondansetron + dexamethasone (*p* = NR) ^b^	−0.9 nausea NRS (0–10) mean diff. at 2 h for ondansetron only (*p* = NR); −2.4 less MME used at 2 h for ondansetron only (*p* = NR) ^b^	I = 0/90 C = 0/90
Judkins and Harmer 1982 [39]	Elective major abdominal surgery	Randomized controlled trial	34 (unknown)	--	PO 10 mg diazepam + IV 5 mg haloperidol; PO 10 mg diazepam + IV 10 mg haloperidol ^d^	PO 10 mg diazepam + IV placebo	No difference in pain VAS (0–100) in haloperidol arms vs. placebo at 24 h (+3.8 median diff., *p* = 0.82)	+ 8.8 additional total MME in haloperidol arms vs. placebo (*p* = NR); −38 to 39 nausea VAS (0–100) median diff. in haloperidol arms vs. placebo (*p* = 0.005)	I = 3/34 C = 1/34
Chronic pain (2 studies)									
Salpeter et al., 2015 [40]	Inpatient palliative care consultation for uncontrolled pain	Retrospective comparative study	43 (67%) 42% cancer)	--	Scheduled PO haloperidol with 2.5–15 mg methadone + PO/IV haloperidol for breakthrough pain (1.5 mg median daily haloperidol dose at 2 weeks)	Scheduled PO haloperidol with PO 2.5–15 mg methadone + PO/IV short-acting opiates for breakthrough pain (0.8 mg median daily haloperidol dose at 2 weeks)	−1.5 pain NRS (0–10) diff. in peak pain scores at 2 weeks (*p* = NR); −16% fewer participants reporting pain ≥7 on pain NRS at 2 weeks (*p* = NR)	+0.5 pain NRS diff. in peak pain scores at 2 weeks in cancer vs. noncancer participants (*p* = NR); baseline median MEDD 79 mg decreased to 6 mg in I arm, 15 mg in C arm (*p* = NR)	I = 0/43 C = 0/43
Raft et al., 1979 [41]	Chronic facial pain	Prospective single-arm trial	16 (unknown)	--	Scheduled PO 2–6 mg/day haloperidol for up to 6 weeks + relaxation therapy, physiotherapy, drug therapy, and counseling	--	All 16 patients felt pain improved ≥65% from baseline on Tourniquet Pain Ratio; 15/16 reported ≥85% from baseline	Decreased paranoia, mania, and social introversion as measured on MMPI (*p* < 0.01 each); increased hypochondriasis in males (*p* < 0.5), depression in females (*p* < 0.01)	I = 2/16 C = 0/16

I indicate intervention arm; C, comparison arm; diff., difference; MME, morphine milligram equivalent; MEDD, morphine-equivalent daily dose; IM, intramuscular; IV, intravenous; PO, oral; NRS, numeric rating scale; VAS, visual analog scale; NR, not reported; NA, no applicable; ED, emergency department; RR, relative risk; MMPI, Minnesota Multiphasic Personality Inventory. ^a^ This is not a complete list of the exclusion criteria of each study. Highlighted here are exclusion criteria that decrease the relevance of the study to patient populations with pain and how many studies assessed QT intervals. All studies excluded patients who were pregnant, had prior reactions to haloperidol, or were children younger than 12 years. ^b^ Estimated values based on available figures using WebPlotDigitizer (https://automeris.io/). Kazemi et al. [37] outcomes were presented on a Likert scale of 0 = no pain, 1 = mild, 2 = moderate, 3 = severe, 4 = worst possible pain. Benevides et al. [38] outcomes used VNSP (verbal pain score 0–10) and VNSN (verbal nausea score 0–10). ^c^ Benevides et al. [38] was three-arm study of ondansetron only, ondansetron with dexamethasone, and ondansetron with dexamethasone and haloperidol; two non-haloperidol arms are presented in the comparison cell. ^d^ Judkins and Harmer was a three-arm study of oral diazepam premedication with IV 5 mg haloperidol, IV 10 mg haloperidol, or placebo that was not further described; two haloperidol arms are presented in the intervention cell.

**Table 3:** Adverse effects reported in haloperidol trials.

Study	Indication	Design	Intervention	Comparison Arm	Dose Used	Participant Size	Adverse Events Reported	Intervention Arm Side Effects	Comparison Arm Side Effects
Honkaniemi et al., 2006 [26]	Acute migraine	Randomized controlled trial	IV haloperido0l + normal saline	IV normal saline alone	5 mg	40	I = 16/40 C = 1/40	9 motor agitation and 9 sedation across 16 participants	1 visual disturbances
McCoy et al., 2020 [27]	Acute headache or migraine	Randomized controlled trial	IV haloperidol	IV placebo	2.5 mg	118	I = 14/118 C = 5/118	4 anxieties 6 restlessness (10%) 2 nausea/vomiting 4 other	4 nausea/vomiting 1 other
Masoumi et al., 2019 [29]	Acute renal colic	Randomized controlled trial	IV morphine 5 mg + IV haloperidol	IV morphine 5 mg + normal saline	5 mg	140	I = 73/140 C = 72/140	34 nausea 36 vomiting 3 extrapyramidal (4.3%)	39 nausea 33 vomiting 0 extrapyramidal
Knudsen-Lachendro et al., 2021 [31]	Nonspecific acute abdominal pain	Retrospective comparative study	IV haloperidol	IV ketorolac 30 mg	5 mg	107	I = 0/107 C = 1/107	None reported	1 mental status change
Moradi et al., 2022 [32]	Acute pain (47% trauma)	Randomized controlled trial	IV haloperidol + 0.3 mg/kg ketamine	IV fentanyl 1 mg/kg	2.5 mg	200	I = 9/200 C = 2/200	5 vomiting 4 emergence reactions ^a^	2 apneas
Gaffigan et al., 2015 [34]	Acute migraine	Randomized controlled trial	IV haloperidol	IV 10 mg metoclopramide	5 mg	64	I = 17/64 C = 14/64	5 sleepiness 10 restlessness (32%) 2 chest pain	9 sleepiness 1 nausea 4 restlessness (12%)
Judkins and Harmer 1982 [39]	Elective major abdominal surgery	Randomized controlled trial	IV haloperidol	IV placebo	5–10 mg	34	I = 3/34 C = 1/34	1 dry mouth 2 stiffness/shaking (0.1%)	1 dry mouth
Raft et al., 1979 [41]	Chronic facial pain	Case series	IV haloperidol	3 groups: * Myofascial * Neuropathic * Unidentified	5 mg	16	I = 2/16 C = 0/16	2 sedation/confusion	--

I, indicate intervention arm, C, comparison arm. Participants may have reported more than one side effect event, and as such, the number of events may be greater than the number of participants with side effects. ^a^ mergence reactions with ketamine use is the experience of positive and negative psychotic symptoms mimicking schizophrenic features.

## Data Availability

Data is contained within the article.

## References

[1] Dold M., Samara M.T., Li C., Tardy M., Leucht S. (2015). Haloperidol versus first-generation antipsychotics for the treatment of schizophrenia and other psychotic disorders. Cochrane Database Syst. Rev..

[2] Gao K., Kemp D.E., Ganocy S.J., Gajwani P., Xia G., Calabrese J.R. (2008). Antipsychotic-induced extrapyramidal side effects in bipolar disorder and schizophrenia: A systematic review. J. Clin. Psychopharmacol..

[3] Granger B., Albu S. (2005). The haloperidol story. Ann. Clin. Psychiatry.

[4] Granger B. (1999). The discovery of haloperidol. Encephale.

[5] Rahman S., Marwaha R. (2023). Haloperidol. StatPearls.

[6] Baranoglu Kilinc Y., Torun I.E., Kilinc E. (2023). D2 dopamine receptor-mediated mechanisms of dopaminergic system modulation in in vivo and in vitro experimental models of migraine. Eur. J. Neurosci..

[7] Bauch E.M., Andreou C., Rausch V.H., Bunzeck N. (2017). Neural Habituation to Painful Stimuli Is Modulated by Dopamine: Evidence from a Pharmacological fMRI Study. Front. Hum. Neurosci..

[8] Lee I.S., Lee B., Park H.J., Olausson H., Enck P., Chae Y. (2015). A new animal model of placebo analgesia: Involvement of the dopaminergic system in reward learning. Sci. Rep..

[9] Déciga-Campos M., Villafán-Gutiérrez R., Espinosa-Juárez J.V., Jaramillo-Morales O.A., López-Muñoz F.J. (2021). Synergistic interaction between haloperidol and gabapentin in a model of neuropathic nociception in rat. Eur. J. Pharmacol..

[10] Espinosa-Juárez J.V., Jaramillo-Morales O.A., López-Muñoz F.J. (2017). Haloperidol Decreases Hyperalgesia and Allodynia Induced by Chronic Constriction Injury. Basic. Clin. Pharmacol. Toxicol..

[11] Kikuchi N., Irifune M., Shimizu Y., Yoshida K., Morita K., Kanematsu T., Morioka N., Nakata Y., Sakai N. (2015). Selective blockade of N-methyl-D-aspartate channels in combination with dopamine receptor antagonism induces loss of the righting reflex in mice, but not immobility. Psychopharmacology.

[12] Leppert W., Okulicz-Kozaryn I., Kaminska E., Szulc M., Mikolajczak P. (2014). Analgesic effects of morphine in combination with adjuvant drugs in rats. Pharmacology.

[13] Mena-Valdés L.C., Blanco-Hernández Y., Espinosa-Juárez J.V., López-Muñoz F.J. (2021). Haloperidol potentiates antinociceptive effects of morphine and disrupt opioid tolerance. Eur. J. Pharmacol..

[14] Petronilho A., Reis G.M., Dias Q.M., Fais R.S., Prado W.A. (2012). Antinociceptive effect of stimulating the zona incerta with glutamate in rats. Pharmacol. Biochem. Behav..

[15] Cooper I., Landersdorfer C.B., St John A.G., Graudins A. (2018). The pharmacokinetics of intranasal droperidol in volunteers characterised via population modelling. SAGE Open Med..

[16] Mauri M.C., Paletta S., Maffini M., Colasanti A., Dragogna F., Di Pace C., Altamura A.C. (2014). Clinical pharmacology of atypical antipsychotics: An update. EXCLI J..

[17] Baldessarini R. (1990). Drugs and the treatment of psychiatric disorders. The Pharmacological Basis of Therapeutics.

[18] Fang J., McKay G., Song J., Remillrd A., Li X., Midha K. (2001). In vitro characterization of the metabolism of haloperidol using recombinant cytochrome p450 enzymes and human liver microsomes. Drug Metab. Dispos..

[19] Murray M. (2006). Role of CYP pharmacogenetics and drug-drug interactions in the efficacy and safety of atypical and other antipsychotic agents. J. Pharm. Pharmacol..

[20] Spina E., de Leon J. (2007). Metabolic drug interactions with newer antipsychotics: A comparative review. Basic. Clin. Pharmacol. Toxicol..

[21] Lawson G.M. (1994). Monitoring of serum haloperidol. Mayo Clin. Proc..

[22] Cobos E.J., Baeyens J.M. (2015). Use of Very-Low-Dose Methadone and Haloperidol for Pain Control in Palliative Care Patients: Are the Sigma-1 Receptors Involved?. J. Palliat. Med..

[23] Richelson E. (1999). Receptor pharmacology of neuroleptics: Relation to clinical effects. J. Clin. Psychiatry.

[24] Bonifazi A., Battiti F.O., Sanchez J., Zaidi S.A., Bow E., Makarova M., Cao J., Shaik A.B., Sulima A., Rice K.C. (2021). Novel Dual-Target μ-Opioid Receptor and Dopamine D3 Receptor Ligands as Potential Nonaddictive Pharmacotherapeutics for Pain Management. J. Med. Chem..

[25] Roldan C.J., Chambers K.A., Paniagua L., Patel S., Cardenas-Turanzas M., Chathampally Y. (2017). Randomized Controlled Double-blind Trial Comparing Haloperidol Combined with Conventional Therapy to Conventional Therapy Alone in Patients with Symptomatic Gastroparesis. Acad. Emerg. Med..

[26] Honkaniemi J., Liimatainen S., Rainesalo S., Sulavuori S. (2006). Haloperidol in the acute treatment of migraine: A randomized, double-blind, placebo-controlled study. Headache.

[27] McCoy J.J., Aldy K., Arnall E., Petersen J. (2020). Treatment of Headache in the Emergency Department: Haloperidol in the Acute Setting (THE-HA Study): A Randomized Clinical Trial. J. Emerg. Med..

[28] Ramirez R., Stalcup P., Croft B., Darracq M.A. (2017). Haloperidol undermining gastroparesis symptoms (HUGS) in the emergency department. Am. J. Emerg. Med..

[29] Masoumi K., Delirrooyfard A., Salehzadeh M. (2019). Comparison of the analgesic effects of haloperidol with or without morphine in patients with acute renal colic: A randomized double-blind clinical trial study. Am. J. Emerg. Med..

[30] Heard K., Bebarta V.S., Hoppe J.A., Monte A.A. (2020). Does administration of haloperidol or ketorolac decrease opioid administration for abdominal pain patients? A retrospective study. Am. J. Emerg. Med..

[31] Knudsen-Lachendro K., Stith K., Vicarel L.A., Harbert B., Fertel B.S. (2021). Study of Haloperidol for Abdominal Pain in the Emergency Department (SHAPE). West. J. Emerg. Med..

[32] Moradi M.M., Moradi M.M., Safaie A., Baratloo A., Payandemehr P. (2022). Sub dissociative dose of ketamine with haloperidol versus fentanyl on pain reduction in patients with acute pain in the emergency department; A randomized clinical trial. Am. J. Emerg. Med..

[33] Afzalimoghaddam M., Edalatifard M., Nejati A., Momeni M., Isavi N., Karimialavijeh E. (2016). Midazolam Plus Haloperidol as Adjuvant Analgesics to Morphine in Opium Dependent Patients: A Randomized Clinical Trial. Curr. Drug Abuse Rev..

[34] Gaffigan M.E., Bruner D.I., Wason C., Pritchard A., Frumkin K. (2015). A Randomized Controlled Trial of Intravenous Haloperidol vs. Intravenous Metoclopramide for Acute Migraine Therapy in the Emergency Department. J. Emerg. Med..

[35] Ali W.I., Hadi E.A.Y., Al-Johar Z.A. (2018). Pain Management by a Combination of Tramadol, Haloperidol and Carbamazepine in Iraqi Burn Patients. Int. J. Med. Res. Health Sci..

[36] Heriwardito A., Manggala S.K., Widhyanti S.I., Aristya L. (2022). Haloperidol vs. Dexamethasone in Lowering Postoperative Nausea and Vomiting and Pain in Adult After Laparoscopy: A Randomized, Double-Blind Study. Bali J. Anesthesiol..

[37] Kazemi A.P., Jowkar T., Amini A., Heydari S.T. (2015). Haloperidol Adjunct with Morphine on Postoperative Pain Management in Opioid-Addicted Patients Undergoing Orthopedic Surgery. Shiraz E-Med. J..

[38] Benevides M.L., de Souza Oliveira S., Aguilar-Nascimento J.E. (2013). Combination of haloperidol, dexamethasone, and ondansetron reduces nausea and pain intensity and morphine consumption after laparoscopic sleeve gastrectomy. Braz. J. Anesthesiol..

[39] Judkins K.C., Harmer M. (1982). Haloperidol as an adjunct analgesic in the management of postoperative pain. Anaesthesia.

[40] Salpeter S.R., Buckley J.S., Buckley N.S., Bruera E. (2015). The use of very-low-dose methadone and haloperidol for pain control in the hospital setting: A preliminary report. J. Palliat. Med..

[41] Raft D., Toomey T., Gregg J.M. (1979). Behavior modification and haloperidol in chronic facial pain. South. Med. J..

[42] Salaffi F., Stancati A., Silvestri C.A., Ciapetti A., Grassi W. (2004). Minimal clinically important changes in chronic musculoskeletal pain intensity measured on a numerical rating scale. Eur. J. Pain.

[43] Lee J.S., Hobden E., Stiell I.G., Wells G.A. (2003). Clinically important change in the visual analog scale after adequate pain control. Acad. Emerg. Med..

[44] Camilleri M., Atieh J. (2021). New Developments in Prokinetic Therapy for Gastric Motility Disorders. Front. Pharmacol..

[45] Thia I., Saluja M. (2021). An update on management of renal colic. Aust. J. Gen. Pract..

[46] Ramsay M.A.E. (2000). Acute postoperative pain management. Bayl. Univ. Med. Cent. Proc..

[47] Sanders D.S., Azmy I.A., Hurlstone D.P. (2006). A New Insight into Non-Specific Abdominal Pain. Ann. R. Coll. Surg. Engl..

[48] McNamee R. (2005). Regression modelling and other methods to control confounding. Occup. Environ. Med..

[49] Frey T.M., Florin T.A., Caruso M., Zhang N., Zhang Y., Mittiga M.R. (2019). Effect of Intranasal Ketamine vs Fentanyl on Pain Reduction for Extremity Injuries in Children. JAMA Pediatr..

[50] Diener H.C., Tassorelli C., Dodick D.W., Silberstein S.D., Lipton R.B., Ashina M., Becker W.J., Ferrari M.D., Goadsby P.J., Pozo-Rosich P. (2019). Guidelines of the International Headache Society for controlled trials of acute treatment of migraine attacks in adults: Fourth edition. Cephalalgia.

[51] Khandeparkar A., Rataboli P.V. (2017). A study of harmful drug–drug interactions due to polypharmacy in hospitalized patients in Goa Medical College. Perspect. Clin. Res..

[52] Emery M.A., Eitan S. (2020). Drug-specific differences in the ability of opioids to manage burn pain. Burns.

[53] Gan T.J., Diemunsch P., Habib A.S., Kovac A., Kranke P., Meyer T.A., Watcha M., Chung F., Angus S., Apfel C.C. (2014). Consensus Guidelines for the Management of Postoperative Nausea and Vomiting. Anesth. Analg..

[54] Chou R., Gordon D.B., de Leon-Casasola O.A., Rosenberg J.M., Bickler S., Brennan T., Carter T., Cassidy C.L., Chittenden E.H., Degenhardt E. (2016). Management of Postoperative Pain: A Clinical Practice Guideline from the American Pain Society, the American Society of Regional Anesthesia and Pain Medicine, and the American Society of Anesthesiologists’ Committee on Regional Anesthesia, Executive Committee, and Administrative Council. J. Pain.

[55] Meyer-Massetti C., Cheng C.M., Sharpe B.A., Meier C.R., Guglielmo B.J. (2010). The FDA extended warning for intravenous haloperidol and torsades de pointes: How should institutions respond?. J. Hosp. Med..

[56] Beach S.R., Gross A.F., Hartney K.E., Taylor J.B., Rundell J.R. (2020). Intravenous haloperidol: A systematic review of side effects and recommendations for clinical use. Gen. Hosp. Psychiatry.

[57] Peabody C.A., Brody D., Warner M.D. (1987). Tardive dyskinesia after low-dose haloperidol. Biol. Psychiatry.

